# Assessing graph-based read mappers against a baseline approach highlights strengths and weaknesses of current methods

**DOI:** 10.1186/s12864-020-6685-y

**Published:** 2020-04-06

**Authors:** Ivar Grytten, Knut D. Rand, Alexander J. Nederbragt, Geir K. Sandve

**Affiliations:** 1 Department of informatics, University of Oslo, Gaustadalleen 23 B, Oslo, 0371 Norway; 2 Department of Mathematics, University of Oslo, Moltke Moes vei 35, Oslo, 0851 Norway; 3 Department of Biosciences, University of Oslo, Blindernvn. 31, Oslo, 0371 Norway

**Keywords:** Graph genomes, Read mapping, Pan-genomics, Reference genomes, Graph-based references, Sequence alignment

## Abstract

**Background:**

Graph-based reference genomes have become popular as they allow read mapping and follow-up analyses in settings where the exact haplotypes underlying a high-throughput sequencing experiment are not precisely known. Two recent papers show that mapping to graph-based reference genomes can improve accuracy as compared to methods using linear references. Both of these methods index the sequences for most paths up to a certain length in the graph in order to enable direct mapping of reads containing common variants. However, the combinatorial explosion of possible paths through nearby variants also leads to a huge search space and an increased chance of false positive alignments to highly variable regions.

**Results:**

We here assess three prominent graph-based read mappers against a hybrid baseline approach that combines an initial path determination with a tuned linear read mapping method. We show, using a previously proposed benchmark, that this simple approach is able to improve overall accuracy of read-mapping to graph-based reference genomes.

**Conclusions:**

Our method is implemented in a tool Two-step Graph Mapper, which is available at https://github.com/uio-bmi/two_step_graph_mapperalong with data and scripts for reproducing the experiments. Our method highlights characteristics of the current generation of graph-based read mappers and shows potential for improvement for future graph-based read mappers.

## Background

As more and more genomes are being sequenced, graph-based reference genomes have become useful for representing and analysing the vast amount of genetic information that is now available [1]. During the last few years, graph-based reference genomes have been used in various next-generation sequencing experiments, such as in variant calling [2, 3], structural variant genotyping [4–6] and peak calling [7]. A key step in many such analysis pipelines is the alignment of raw sequencing reads to the reference [8]. Recently, two tools for mapping reads to graph-based reference genomes have been proposed – *vg* [3] and a tool created by *Seven Bridges* [9] (from here on we refer to this tool as Seven Bridges). Both show improved mapping accuracy compared to the linear reference-based method Burrows-Wheeler Aligner MEM (BWA-MEM) [10]. While *vg* indexes all paths up to a certain length in the graph – a tedious process that takes more than a day for a human whole-genome graph – Seven Bridges uses a faster approach in which only short kmers (21 base pair sequences at 7 base pair intervals) are indexed. This enables indexing of a human whole-genome graph in only minutes. A third method for mapping reads to graph-based references is Hisat 2, which uses a Hierarchical Graph Full-text index in Minute space (FM) index [11]. As complex graphs containing many genetic variants can result in long indexing time as well as poor mapping accuracy [3], existing graph-based read mappers ignore the most complex regions in the graph when indexing the graph. Another strategy for reducing graph complexity is to limit the number of genetic variants that are included in the graph in the first place [12]. Some have also proposed to not use graphs, but instead improve the current linear reference genome [13].

There currently exists no comparison of the mapping accuracy of *vg*, Seven Bridges and Hisat 2. Furthermore, there exists no study on how these tools perform compared to linear mapping approaches tuned for accuracy and not speed, or to simpler schemes for graph-based read mapping. We here present a hybrid graph-mapping approach and use this as a baseline to highlight strengths and potential for improvement for the current generation of graph-based mapping approaches that are able to map reads to graphs built from a linear reference genome and a set of genetic variants. We compare *vg*, Seven Bridges and Hisat 2 to a tuned linear mapping approach, and to our two-step approach, and show that graph-based read mapping can be improved by separating the problem into rough path estimation and subsequent mapping of each individual read to this estimated path.

## Results

In the following, we assess graph-mappers by looking at *vg*, Seven Bridges and Hisat 2. All assessments are done by following the approach that *vg* and Seven Bridges used for evaluating their tools [9]. We simulate single-end reads with read length 150 bases from the whole genome of an Ashkenazi Jewish male NA24385, sequenced by the Genome in a Bottle Consortium [14] (see “Methods” section). We simulate uniformly across the genome, and some reads will naturally be simulated from segments containing non-reference alleles (about 10.6% of the reads). We refer to these as *reads with variants*. Reads that are simulated from segments identical to the linear reference genome (hg19) will be referred to as *reads without variants*. Mapping accuracies are compared using receiver operating characteristic (ROC) curves parameterized by the mapping quality (MAPQ) of all the simulated reads, where each dot in the plot shows the recall and error rate for reads with at least the corresponding MAPQ. Scripts and data for generating the figures in this section are provided at https://github.com/uio-bmi/two_step_graph_mapper.

### *vg* outperforms seven bridges and hisat 2 on previously proposed benchmarks

**Fig. 1:**
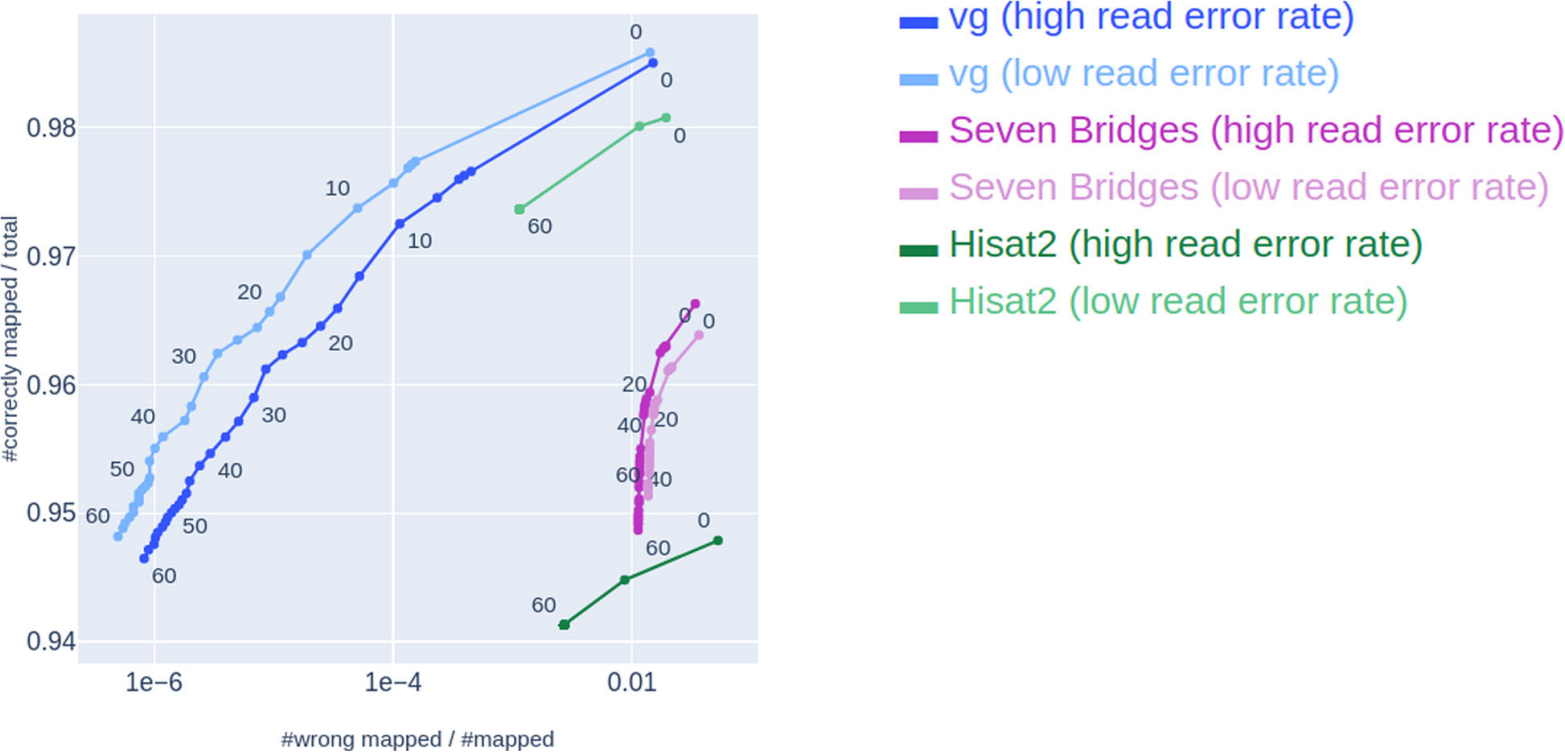
Comparison of existing graph-based read mappers. Comparison of mapping accuracy on reads mapped by *vg*, Seven Bridges and Hisat 2 by ROC-plots parameterized by the MAPQ of reads simulated with high read error rate (substitution rate 1% and indel rate 0.2%) and low read error rate (substitution rate 0.26% and indel rate 0.01%). Each dot represents a MAPQ cut-off, and numbers next to dots specify the cut-off at a given dot

In Fig. 1, we compare the mapping accuracy of *vg*, Seven Bridges and Hisat 2 on 40 million simulated reads, using two different error rates when simulating the reads – 1% substitution rate and 0.2% indel rate, as used by *vg* in [3] (referred to as high read error rate) and with a lower error rate of 0.26% substitution rate and 0.01% substitution rate, which is similar to the error rate used by Seven Bridges in their evaluation [9]. *vg* performs better than both Seven Bridges and Hisat 2 on both error rates. From here on, we thus focus on *vg* when discussing capabilities and limitations of the current generation of graph-based mapping approaches, and use simulated reads with 1% substitution rate and 0.2% indel rate (as used by *vg* in their evaluation).

### Part of the performance difference between graph-based and linear methods can be attributed to method tuning

**Fig. 2:**
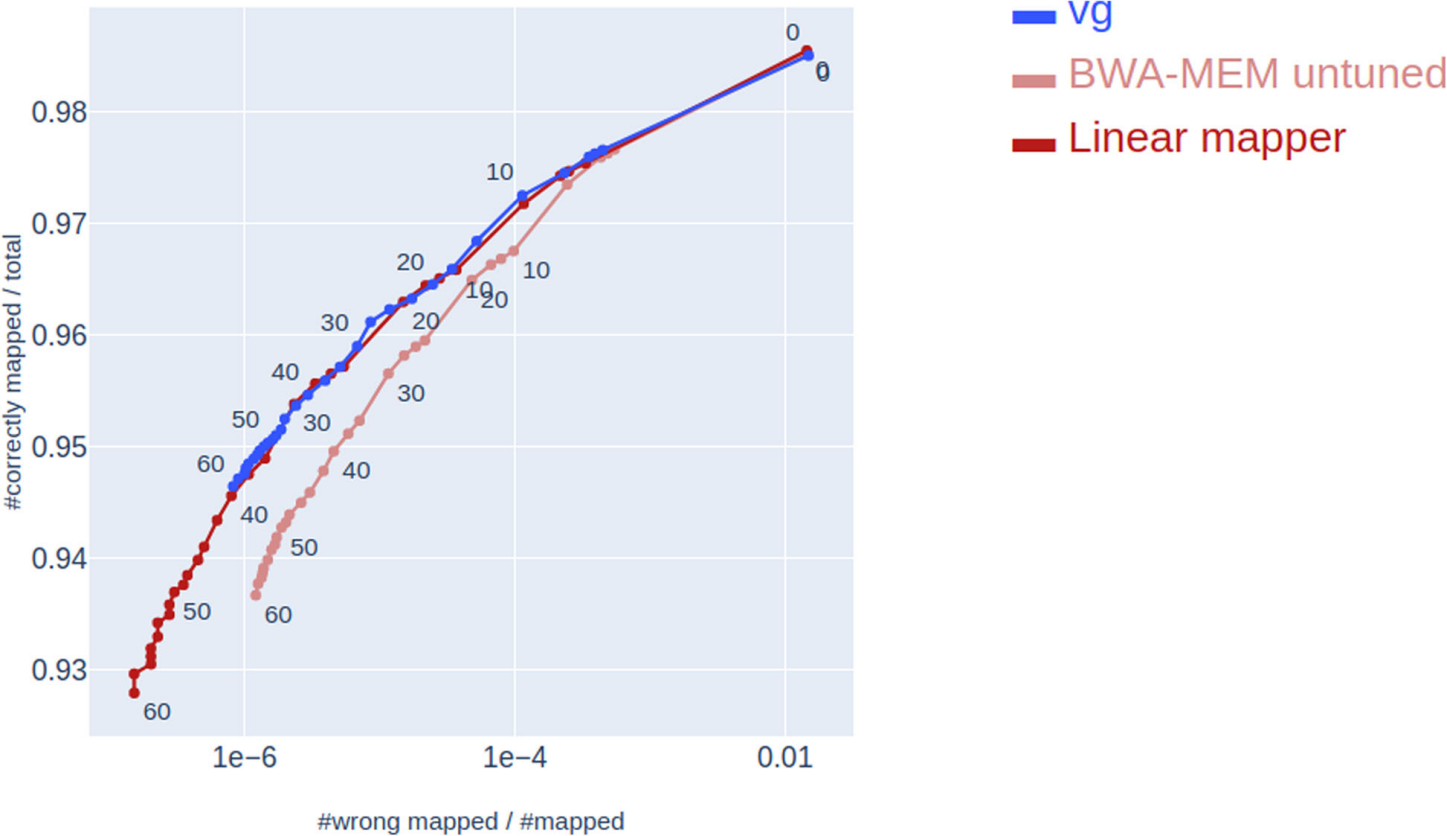
Comparison of vg and tuned linear mapping. Comparison of the mapping accuracies of the linear mapper, *vg* and untuned BWA-MEM (running with default parameters)

As shown in Fig. 2, *vg* performs better than BWA-MEM when BWA-MEM is run with default parameters. However, BWA-MEM is by default tuned for speed and not for maximum accuracy. By tuning BWA-MEM and adjusting the MAPQ scores by also running Minimap 2 (see “Methods” section), BWA-MEM goes from performing worse than *vg* on all reads to be performing about as well as *vg* while still spending less than half the time of vg at mapping the same reads (Table 1). From here on, we use this tuned version of BWA-MEM, referred to as *linear mapper*, when comparing graph-based and linear mapping approaches.

**Table 1:** Run times for the different methods, showing the time spent on processing 576 million reads using 24 computing threads

**Linear mapper**	**12h 51m**
- BWA-MEM (tuned)	7h1m
- Minimap (tuned)	4h40m
- Merging alignments	1h10m
**Two-step approach**	**24h50m**
- Initial rough mapping	6h8m
- Predict path through graph and indexing the path with BWA-MEM	1h21m
- Running linear mapper on path	12h51m
- Post-processing alignments (including conversion to linear reference genome coordinates)	4h30m
**vg**	**28h52m**

Total time is shown in bold text with the time spent for each substep listed below

### Graph-based mapping results in higher accuracy on reads with variants, but lower accuracy on reads without variants

**Fig. 3:**
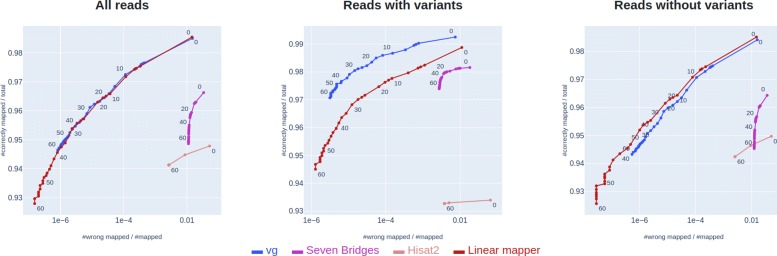
Comparison of the existing graph-based mappers and linear mapping. Comparison of the mapping accuracies of *vg*, Seven Bridges, Hisat 2 and linear mapping

As seen in Fig. 3, *vg* achieves markedly higher accuracy on reads with variants than the linear mapper. However, as also noted in [3], the mapping accuracy of *vg* is lower than the linear mapper on reads that do not contain variants. As a result of this, *vg* ends up not performing better than the linear mapper when assessed on the full set of reads.

### Re-aligning the reads to an estimated linear path through the graph improves accuracy

**Fig. 4:**
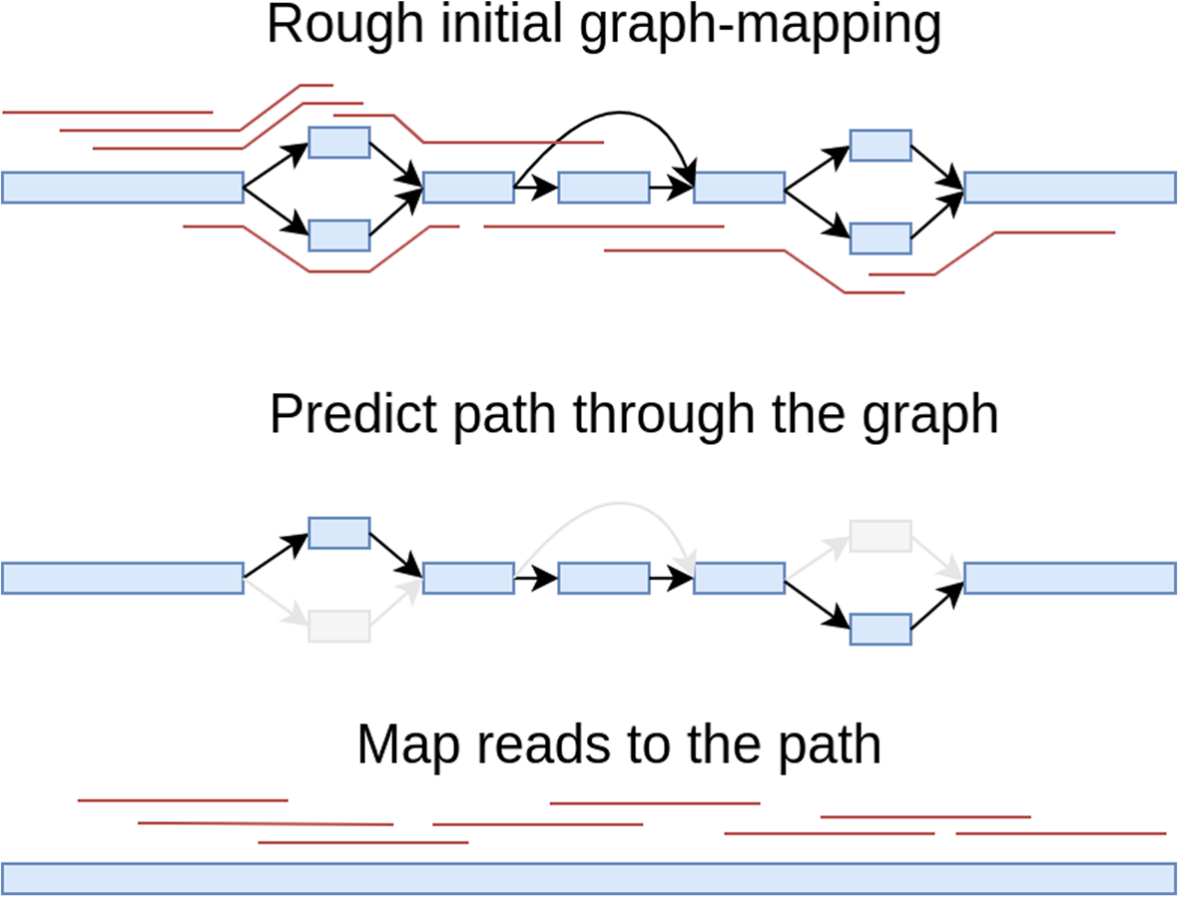
Illustration of the two-step approach to mapping reads to a graph-based reference genome. Top: Reads (red) are first roughly mapped to the graph-based reference genome (nodes represented in blue; edges represented as black arrows). Middle: a path is predicted through the graph depending on where most of the reads map, (parts of the graph no longer included in transparent color). Bottom: in the second step, reads are mapped to the linear path using a linear read mapper

**Fig. 5:**
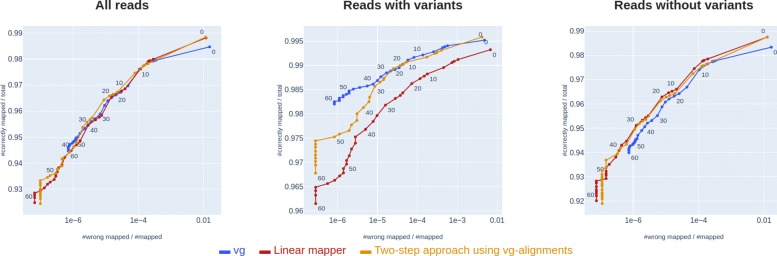
Two-step approach using vg.: Mapping accuracy on 32 million simulated reads from chromosome 20, 21 and 22, showing *vg*, the linear mapper and a two-step approach using *vg* alignments to initially predict a path through the graph and then re-aligning the reads to this path using the linear mapper

We find that using the initial graph alignments to predict a linear path through the graph, and then re-aligning all the reads to this linear path using the linear mapper increases mapping accuracy. This idea is illustrated in Fig. 4, and in Fig. 5 we show the benchmarking results of this approach when using vg to do initial graph mapping. As seen in Fig. 5, this two-step approach performs almost as well as *vg* on reads containing variants – except for reads with high MAPQ, where the method performs slightly worse – and clearly better than *vg* on reads not containing variants, resulting in slightly better overall performance on all reads.

### A two-step approach using an initial rough path estimation is sufficient to improve mapping accuracy

The results from the previous section indicate that the *vg* mapping accuracy may be improved (especially for reads not containing variants) by predicting a path and re-aligning all the reads to this path using the linear mapper. We argue that this idea works as long as we are able to predict an approximate path in the first step. We suggest that the path-prediction in itself can be achieved by initial rough graph-mapping, and as an example, we use an initial rough graph-mapping method where all the reads first are aligned to the linear reference genome and then subsequently locally fitted to the graph. A proof-of-concept implementation of this method is provided in the Python package Rough Graph Mapper (https://github.com/uio-bmi/rough_graph_mapper).

**Fig. 6:**
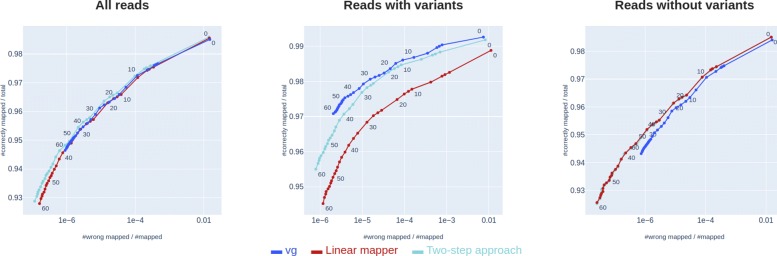
Two-step approach using an initial rough graph mapper. Comparison of mapping accuracies of the two-step approach using an initial rough graph mapper, *vg* and linear mapper. The three methods are run on 576 million reads simulated from the whole genome

**Fig. 7:**
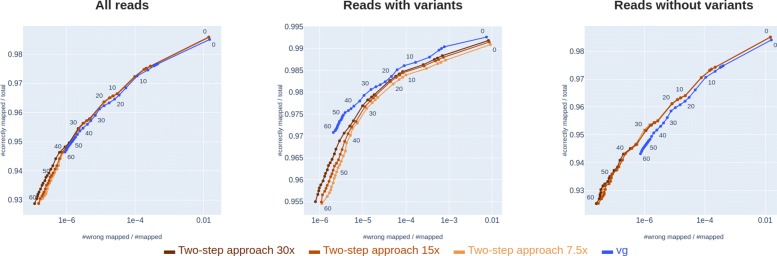
Two-step approach on different read depths. Comparison of the two-step approach on different read depths (7.5x, 15x and 30x) and vg

As seen in Fig. 6, the use of this method in the first step of the two-step approach leads to better mapping accuracy than *vg* for non-variant reads, and almost as good accuracy as *vg* on variant-reads. This two-step approach benefits from high read depth in order to better estimate a path through the graph. The experiment shown in Fig. 6 uses on average read depth of 30. The results of the same experiment run with read depth 15 and 7.5 are shown in Fig. 7. As seen in Fig. 7, the two-step approach performs worse on reads with variants when the read depth is lowered.

Table 1 shows the time used by the different methods, showing that the total time spent by the two-step approach is less than the time used by vg. Furthermore, since the approach only relies on an initial rough mapping that does not rely on a graph index (like the one used by vg) we argue that this two-step approach is a promising direction for computationally efficient graph-based read mapping. Our two-step approach is implemented in a tool Two-step Graph Mapper, which is available at https://github.com/uio-bmi/two_step_graph_mapper.

We also investigate the accuracy of variant calling and genotyping by Graphtyper when using reads mapped by vg, the linear mapper and the two-step approach. We do this by mapping short reads sequenced from the NA24385 individual. We map these reads with vg, the linear mapper and the two-step approach, and run Graphtyper on the three sets of alignments (see “Methods” section). We compare the variants discovered and genotyped by Graphtyper to a set of high-confidence variants for NA24385. Table 2 shows the recall and precision for each method. *vg* has the highest recall but the lowest precision, and the linear mapper has the lowest recall but the highest precision. However, the differences between the methods are minimal.

**Table 2:** Precision and recall when running Graphtyper with reads mapped by the different methods

	Linear mapper	Two-step approach	vg
Indels recall	71.30%	72.21%	72.31%
Indels precision	94.30%	94.30%	94.14%
SNPs recall	94.64%	95.85%	96.21%
SNPs precision	99.35%	99.27%	98.31%

## Discussion

We observe higher accuracy for *vg* than Seven Bridges and Hisat 2 in our comparisons. These three methods all perform worse than linear mapping on reads not containing variants, and a tuned version of BWA-MEM achieves about the same accuracy as *vg* on the full set of reads. We are unsure why Hisat 2 performs worse than vg, but to our knowledge, Hisat 2 is primarily used for RNA and not DNA sequencing reads. We hypothesise that Seven Bridges performs worse than vg because it is using a much simpler index, containing only a subset of all kmers in the graph. We further show that a two-step approach of predicting a path through the graph and mapping to this path using the linear mapper results in higher accuracy on all reads, even when using a rough graph-mapper for the initial prediction of the path. Our two-step approach achieves almost the same accuracy as *vg* on reads containing variants and slightly higher accuracy than *vg* on reads not containing variants (which contribute to about 90% of the simulated reads). We believe this is because the method is able to leverage the information from the full read set mapped in the first step, and also because the use of a predicted path limits the search space dramatically in the final mapping.

While our proposed method does not improve read mapping for reads containing variants – which in many cases are the most interesting reads – it is able to achieve about the same accuracy as vg using a simpler approach and without the lost accuracy on reads not containing variants. It is worth noting that the difference in accuracy between the linear mapper and the graph-based approaches is small compared to the difference in accuracy between the graph-based methods and the tuned linear approach (BWA-MEM + Minimap 2). This shows how important tuning can be for mapping accuracy, and that both tuning and run time should be considered when comparing read mappers. The small differences in accuracy between the different methods is further demonstrated by the small difference in variant detection accuracy (Table 2).

Read alignment serves as an intermediate step for several distinct investigations. The aligned reads may be used as input for variant callers in order to determine genotypes or somatic mutations, for peak callers to determine locations of epigenetic modifications or protein binding to DNA, and for transcriptome analysis methods to quantify differential gene expression or alternative splicing. The consequences of different categories of mis-mapped reads (e.g. reads originating from genomic regions of high or low variation) may vary between these settings. As future work, it would be interesting to explore how the mis-mapping profiles of the different approaches affect the following analysis step for each such setting.

We have shown one implementation of how reads can be mapped in the first step of the two-step approach. This method maps each read to the linear reference genome first and then locally fits each read to the graph. A variant of this method that probably would give better results would be to have the linear mapper report the *n* best hits for each read, locally align each of those to the graph, and pick the alignment with highest graph alignment score. As future work, we also believe it could be interesting to use other graph-based mapping methods that sacrifice accuracy for speed in the first step in the two-step mapping approach. An idea for such a method could be a graph-generalization of minimizer-based mapping methods such as minimap [15].

The method we use for initial rough path prediction is fairly simple and naive, but illustrates the point. As future work, it would be interesting to implement more sophisticated path prediction algorithms, e.g. including haplotype information or correlations between variants in the graph. We note that our two-step approach only performs well when there are sufficient reads for predicting the path (i.e. high enough coverage), and that accuracy drops with lower coverage (Fig. 7). With coverage close to 0 we expect the accuracy to drop down to that of a linear sequence aligner, since our path prediction algorithm defaults to the linear reference genome path when there are not enough reads covering a variant. Our current implementation predicts only one path through the graph, but in reality, reads coming from a diploid individual will follow two paths. It should be trivial to instead estimate two paths in the first step of our two-step approach, and align reads to both paths in the final step.

For linear reference genomes, the sole objective of mapping is to align reads back to the genomic locations they originate from. In contrast, mapping against graph-based reference genomes can serve a dual purpose: estimating the underlying haplotypes (two paths through the graph) and correctly placing each read along these haplotype paths. The driving idea of our two-step approach is to separate these as two different algorithmic problems. This allows a rough mapping approach to be used initially for estimating the haplotype and thus limit the search space for a subsequent step of placing reads along this path using any linear mapper. It is important to note that although the path-estimation in the first step of the two-step approach implicitly estimates variants present in the graph, the intention of this step is not to do variant calling – instead variant calling can be performed as a follow-up step based on the aligned reads.

## Conclusions

We have here proposed a hybrid baseline approach for graph-based read mapping that combines an initial path determination with a tuned linear read mapping method. By comparing three prominent graph-based read mappers to this novel baseline, we find that part of the accuracy gains observed in recent comparisons of graph-based and linear mappers can be attributed to method tuning. Nonetheless, when focusing on reads containing variants (as compared to the linear reference genome), we observe markedly improved accuracy of the graph-based mapper *vg* as compared to mapping to a linear reference using a tuned version of BWA-MEM. Two other graph-based mappers, Seven Bridges and Hisat 2, attain markedly lower mapping accuracy than *vg* in our benchmarks, and do not improve on the linear mapper even on the regions containing variants. By employing *vg* for initial path determination in our proposed two-step approach, we improve on the performance of *vg* used in isolation. Furthermore, even when using a quick, rough mapper for the initial step, our two-step approach performs comparably to the use of *vg* in isolation. In addition to serving as a baseline for highlighting characteristics of the current generation of graph-based read mappers, we thus believe that our two-step approach represents a promising alternative direction for computationally efficient graph-based read mapping.

## Methods

### Assessment of mapping methods

We compared *vg*, Seven Bridges and Hisat 2, which to our knowledge are the main methods for mapping reads to a graph-based reference genome, when considering graphs built from a linear reference genome and a set of genetic variants. We considered to include running BWA-MEM on an index created by CHOP [16], which is a tool for indexing paths through known haplotypes in the graph, but we were unable to build the CHOP index for a whole human genome human graph in reasonable run-time. We also considered to include PanVC [17] in the comparison, but also this method does not seem to scale to a whole human genome, for which it takes weeks to run on [17]. All evaluations were run with vg version 1.19.0, BWA-MEM version 2.0pre1, Minimap 2 version 2.13, Seven Bridges graph aligner bpa-0.9.1.1-3 and Hisat 2.1.0. We ran vg and Seven Bridges with default parameters and Hisat 2 with the –no-spliced-alignment option. When running BWA-MEM, we tuned it and used Minimap in order to adjust the MAPQ scores returned by BWA-MEM (see the next section for details).

The benchmark we ran is similar to the one performed by vg in [3] and the one by Seven Bridges in [9], except that we only simulated single-end reads. We also follow the benchmarking setup of vg in terms of defining reads as correctly mapped when mapped within 150 base pairs of the true location. In all analyses, we used a graph built with vg using variants from the 1000 Genomes Project having allele frequency > 1% (about 14 million variants), since this graph gave the best results for vg and seems to be similar to the graph that Seven Bridges used in their evaluation, which contained 15.8 million variants (mostly from the 1000 genomes project).

We have created a Docker image with all scripts and exact software with dependencies necessary for re-running the benchmarks. This image along with instructions on how to re-run the analysis are available from the readme page at https://github.com/uio-bmi/two_step_graph_mapper.

When comparing variant calling performance using reads mapped by the different methods, we used publicly available reads sequenced from the whole genome of the NA24385 individual, available as a bam file at https://ftp://ftp-trace.ncbi.nlm.nih.gov/giab/ftp/data/AshkenazimTrio/HG002_NA24385_son/NIST_HiSeq_HG002_Homogeneity-10953946/HG002Run01-11419412/HG002run1_S1.bam. We converted the BAM-file to fasta format using the *samtools fasta tool* and downsampled the fasta to half the number of reads by choosing every second read. Variants were called on the whole human genome using Graphtyper version 2.0 and detected variants were compared against high confidence variant calls from https://ftp://ftp-trace.ncbi.nlm.nih.gov/giab/ftp/release/AshkenazimTrio/HG002_NA24385_son/NISTv3.3.2/GRCh37/HG002_GRCh37_GIAB_highconf_CG-IllFB-IllGATKHC-Ion-10X-SOLID_CHROM1-22_v.3.3.2_highconf_triophased.vcf.gzusing Hap.py version 0.3.10-8. Code for reproducing the variant calling experiment using this data is available at https://github.com/uio-bmi/variant_calling_benchmarks_two_step_mapper.

### Tuning BWA-MEM performance

We observe worse performance by BWA-MEM with default parameters compared to vg, even on reads not containing variants. This has also been noted in [3] where vg running on a linear reference genome is shown to outperform BWA-MEM running on the same reference. From our experience, a cause of this is that BWA-MEM is tuned for speed (not only performance), mainly by not trying to align shorter "chains" by default. This makes BWA-MEM miss a lot of suboptimal alignments (when they exist), which in turn makes it overestimate the MAPQ score. Changing the -D parameter of BWA-MEM to a low number partly solves this, by telling BWA-MEM to also try to align shorter chains. However, after such tuning, there are still cases where BWA-MEM fails to find suboptimal alignments. This typically happens when all the longest chains cover a sequencing error in the read. We did not find a way of tuning BWA-MEM to consider shorter chains in such cases, but we found that Minimap 2 (even though it generally performs worse than BWA-MEM on short reads) in most cases was able to find all suboptimal alignments and use that to correctly assign low MAPQ scores when reads were multimapping. Thus, we chose to also run Minimap 2 on the same reads and for every read, simply selecting the MAPQ score chosen by Minimap 2 when Minimap 2 assigned a lower MAPQ score than BWA-MEM.

We did not tune vg in order to try to improve its mapping accuracy, since it seems that vg with default parameters is already tuned to perform well, and is slower than running both tuned BWA-MEM and tuned Minimap 2.

### Two-step graph mapping

#### Step 1: estimating a path through the graph

In order to predict a path through the graph, some initial graph-alignments are needed. We have proposed a simple way for performing this initial mapping, which is explained more in detail in the next section. In this first step, we use the rough graph alignments to predict a path through the graph. We do this by greedily traversing the graph by always following the edges with most reads aligned to them, but requiring a minimum number of reads on edges diverging from the linear reference path. We then extract the sequence of this path, and use BWA (bwa index with default parameters) to index it.

#### Step 2: aligning the reads

In this step, we simply align all the reads to the indexed path using the linear mapper (which first maps all the reads with BWA-MEM and then again maps all the reads with Minimap 2). In the rare cases where a read maps with higher score during the rough initial alignment than to the predicted path in the second pass, the initial rough alignment is chosen. The reads mapped with the linear mapper to the predicted path have coordinates relative to this path, differing from the linear reference genome (e.g. hg19) coordinates. We have implemented a simple method for translating the coordinates back to linear reference genome coordinates (similar to vg annotate) that simply moves the alignments to the graph and finds the closest position on the linear reference path going through the graph.

### Rough mapping of reads to the graph

First, we map all the reads to the linear reference genome using BWA-MEM without any tuning. We then move these reads to their corresponding position in the graph using the linear reference path through the graph and the alignment information to that path from the SAM-file.

## Abbreviations

BWA-MEM: Burrows-wheeler aligner MEM FM index: Full-text index in minute space index ROC curve: Receiver operating characteristic curve MAPQ: Mapping quality
